# Supplementary material for: Thymoquinone-protoflavone hybrid molecules as potential antitumor agents
Source: PLoS One. 2024 Jan 25;19(1):e0291567. doi: 10.1371/journal.pone.0291567 (PMC10810434; doi:10.1371/journal.pone.0291567)

**Supporting information for**

**Thymoquinone-Protoflavone Hybrid Molecules as Potential Antitumor Agents**

Sara H. H. Ahmed^1,#^, Bizhar A. Tayeb^2^, Tímea Gonda^1^, Gábor Girst^1^, Kornél Szőri^1^, Róbert Berkecz^3^, István Zupkó^2^**,** Renáta Minorics^2^, Attila Hunyadi^1,4,^*

*^1^Institute of Pharmacognosy, University of Szeged, H-6720 Szeged, Hungary; ^2^ Institute of Pharmacodynamics and Biopharmacy, University of Szeged, H-6720 Szeged, Hungary, ^3^ Institute of Pharmaceutical Analysis, University of Szeged, 4, 6720 Szeged, Hungary, ^4^ Interdisciplinary Centre of Natural Products, University of Szeged, H-6720 Szeged, Hungary*

***** Correspondence: hunyadi.attila@szte.hu; Tel.: +36-62545557

^#^ On leave from the Department of Pharmaceutical Chemistry, Faculty of Pharmacy, University of Khartoum, 11111 Khartoum, Sudan.

**Table of contents**

Figure S1. HRMS spectrum of compound 3

Figure S2. HRMS spectrum of compound 4

Figure S3. HRMS spectrum of compound 10

Figure S4. HRMS spectrum of compound **11**

Figure S5. HRMS spectrum of compound 12
Figure S6. HRMS spectrum of compound 13
Figure S7. HRMS spectrum of compound 14
Figure S8. HRMS spectrum of compound 15
Figure S9. HRMS spectrum of compound 16

Figure S10. HRMS spectrum of compound 17

Figure S11. ^1^H-NMR spectrum of compound 2

Figure S12. ^13^C-NMR spectrum of compound **2**

Figure S13. edHSQC spectrum of compound 2

Figure S14. HMBC spectrum of compound 2

Figure S15. COSY spectrum of compound 2

Figure S16. ^1^H-NMR spectrum of compound 3

Figure S17. ^13^C-NMR spectrum of compound **3**

Figure S18. edHSQC spectrum of compound 3

Figure S19. HMBC spectrum of compound 3

Figure S20. COSY spectrum of compound 3

Figure S21. ^1^H-NMR spectrum of compound 4
Figure S22. ^13^C-NMR spectrum of compound 4

Figure S23. edHSQC spectrum of compound 4

Figure S24. HMBC spectrum of compound **4**

Figure S25. COSY spectrum of compound 4
Figure S26. ^1^H-NMR spectrum of compound 10
Figure S27. ^13^C-NMR spectrum of compound 10

Figure S28. edHSQC spectrum of compound 10

Figure S29. HMBC spectrum of compound 10

Figure S30. COSY spectrum of compound 10
Figure S31. ^1^H-NMR spectrum of compound 11
Figure S32. ^13^C-NMR spectrum of compound 11

Figure S33. edHSQC spectrum of compound 11

Figure S34. HMBC spectrum of compound 11

Figure S35. COSY spectrum of compound 11
Figure S36. ^1^H-NMR spectrum of compound 12
Figure S37. ^13^C-NMR spectrum of compound 12

Figure S38. edHSQC spectrum of compound 12

Figure S39. HMBC spectrum of compound 12

Figure S40. COSY spectrum of compound 12

Figure S41. ^1^H-NMR spectrum of compound 13

Figure S42. ^13^C-NMR spectrum of compound 13

Figure S43. edHSQC spectrum of compound 13

Figure S44. HMBC spectrum of compound 13

Figure S45. COSY spectrum of compound 13
Figure S46. ^1^H-NMR spectrum of compound 14

Figure S47. ^13^C-NMR spectrum of compound 14

Figure S48. edHSQC spectrum of compound 14

Figure S49. HMBC spectrum of compound 14

Figure S50. COSY spectrum of compound 14

Figure S51. ^1^H-NMR spectrum of compound 15

Figure S52. ^13^C-NMR spectrum of compound 15

Figure S53. edHSQC spectrum of compound 15

Figure S54. HMBC spectrum of compound 15

Figure S55. COSY spectrum of compound 15

Figure S56. ^1^H-NMR spectrum of compound 16

Figure S57. ^13^C-NMR spectrum of compound 16

Figure S58. edHSQC spectrum of compound 16

Figure S59. HMBC spectrum of compound 16

Figure S60. COSY spectrum of compound 16

Figure S61. ^1^H-NMR spectrum of compound 17

Figure S62. ^13^C-NMR spectrum of compound 17

Figure S63. edHSQC spectrum of compound 17

Figure S64. HMBC spectrum of compound 17

Figure S65. COSY spectrum of compound 17

Figure S1. HRMS spectrum of compound 3

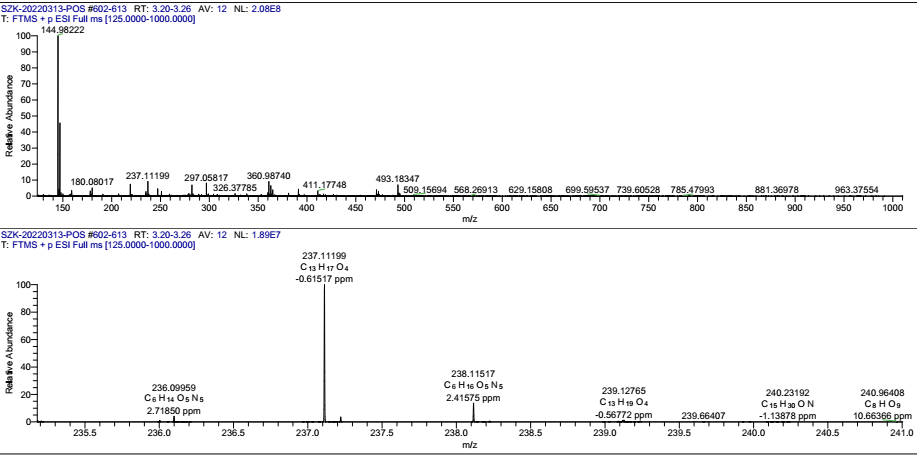


Figure S2. HRMS spectrum of compound 4


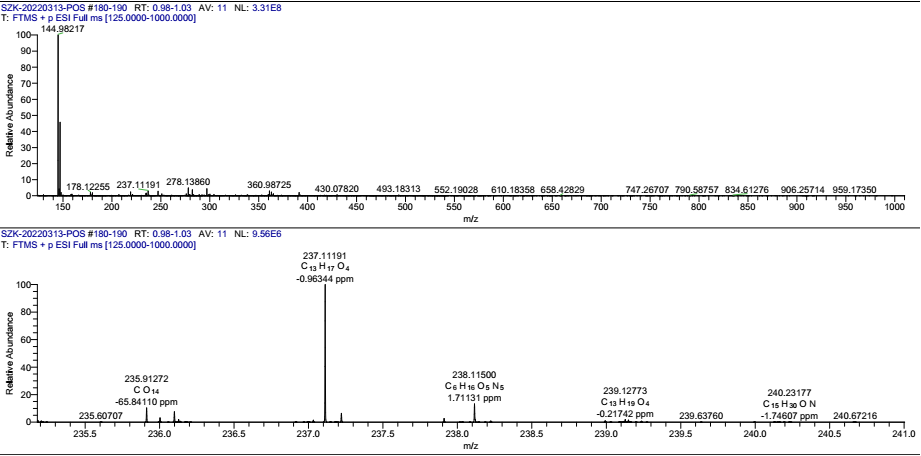


Figure S3. HRMS spectrum of compound 10


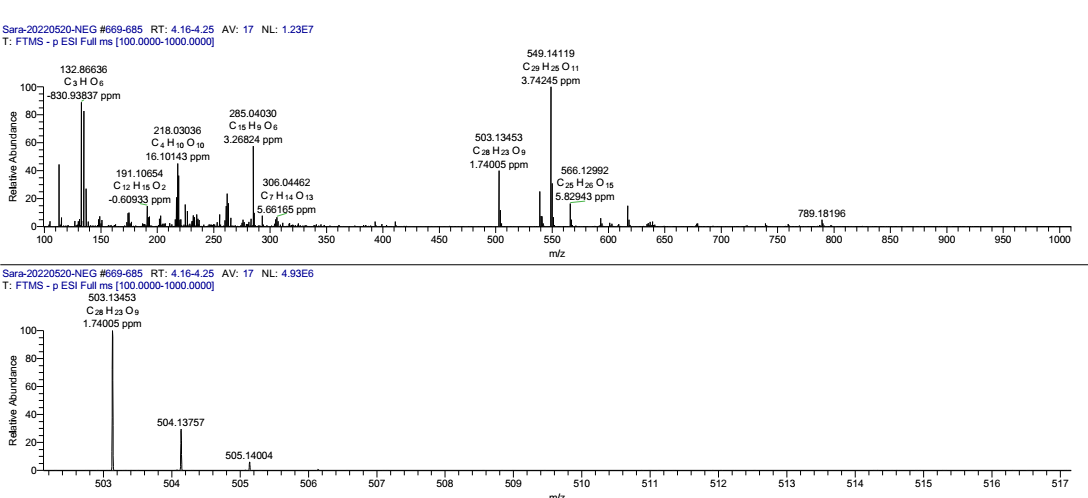


Figure S4. HRMS spectrum of compound 11


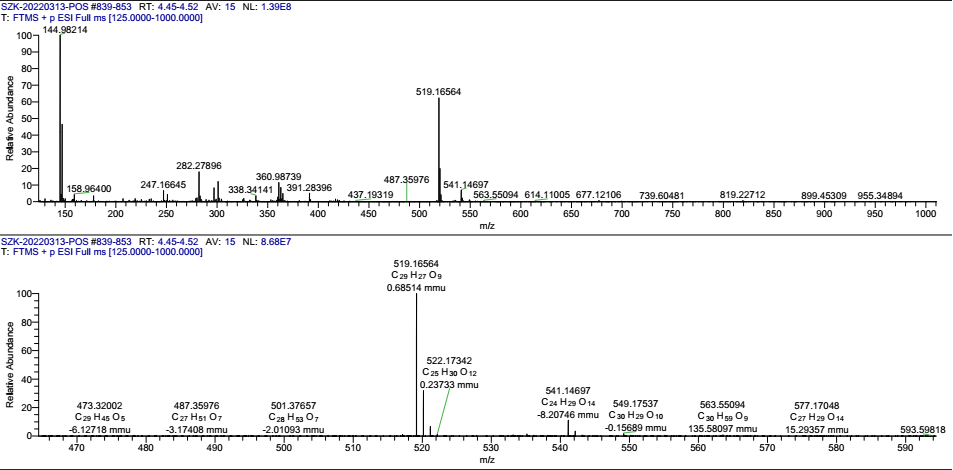


Figure S5. HRMS spectrum of compound 12


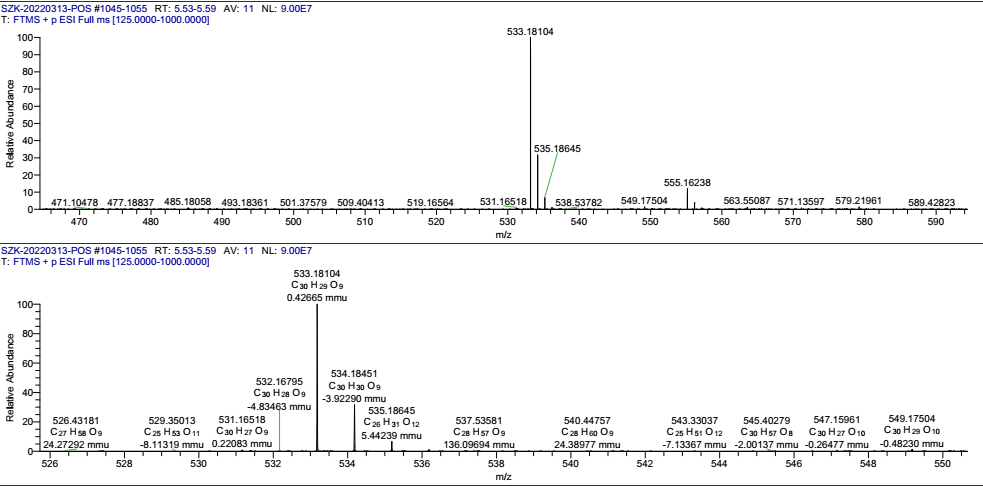


Figure S6. HRMS spectrum of compound 13


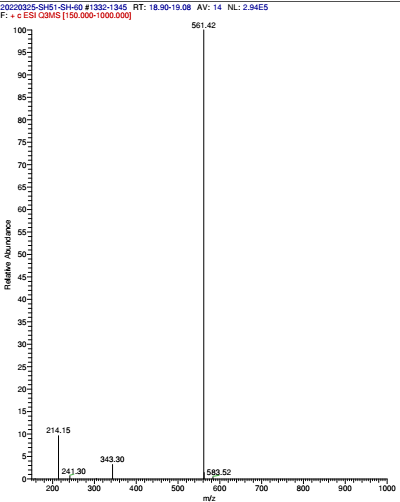


Figure S7. HRMS spectrum of compound 14


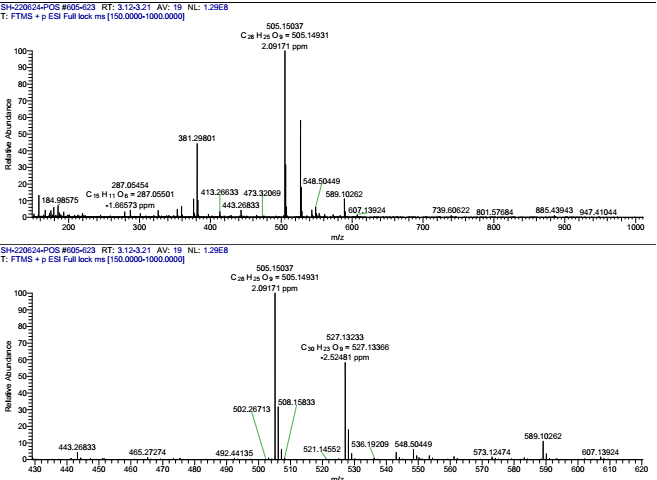


Figure S8. HRMS spectrum of compound 15


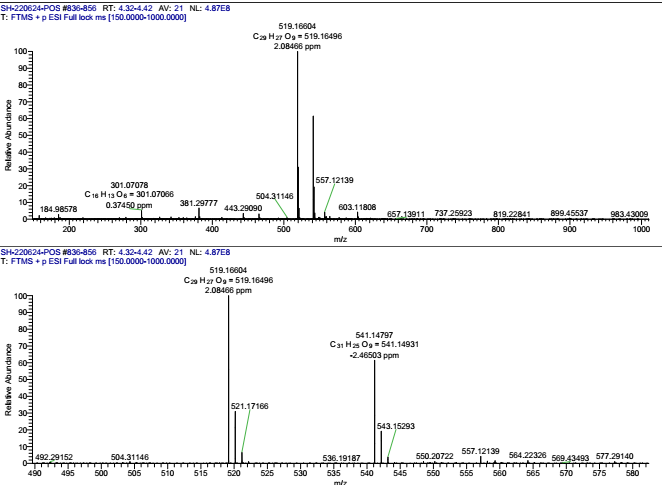


Figure S9. HRMS spectrum of compound 16


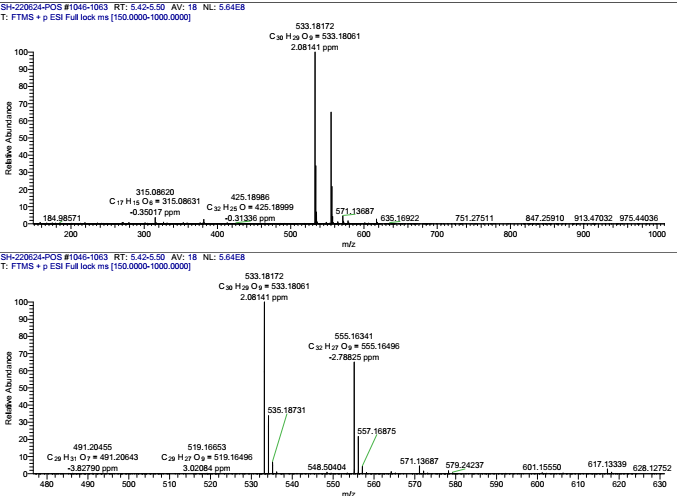


Figure S10. HRMS spectrum of compound 17


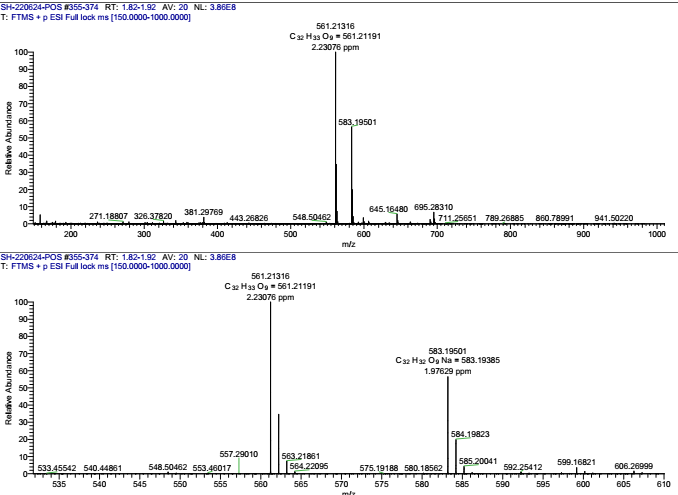

Figure S11. ^1^H-NMR spectrum of compound 2
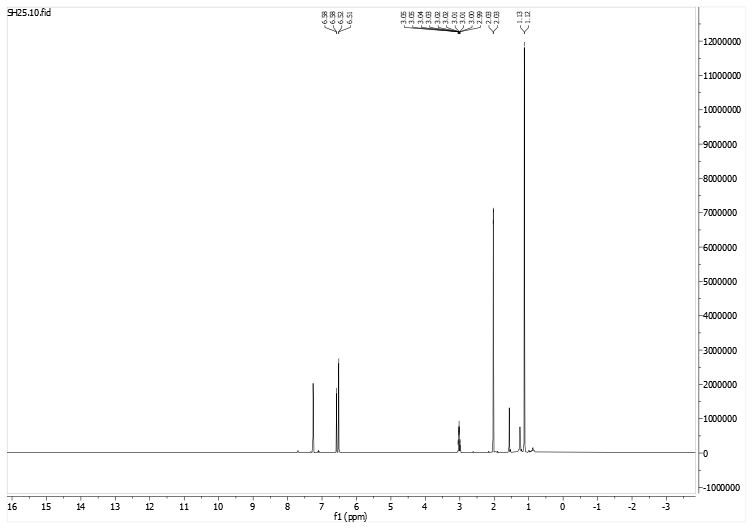


**Figure** S12. ^13^C-NMR spectrum of compound 2
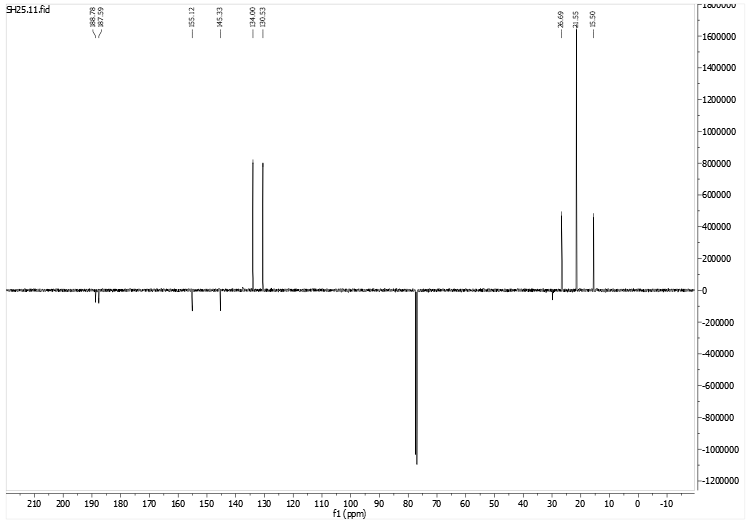


Figure S13. edHSQC spectrum of compound 2
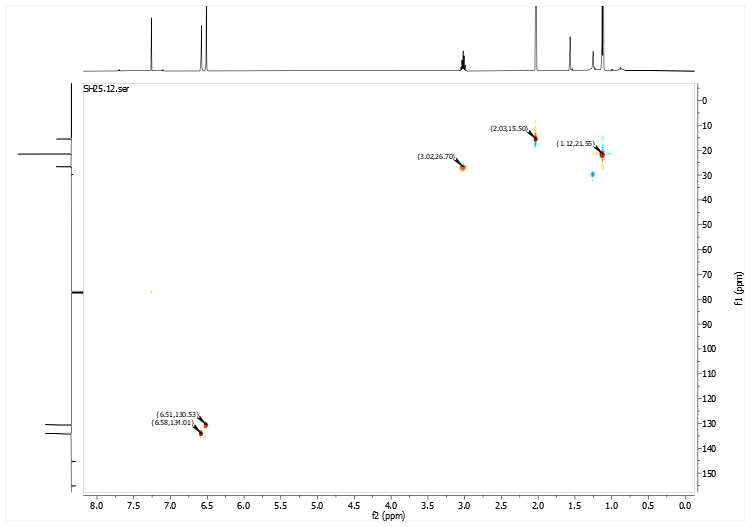


Figure S14. HMBC spectrum of compound 2
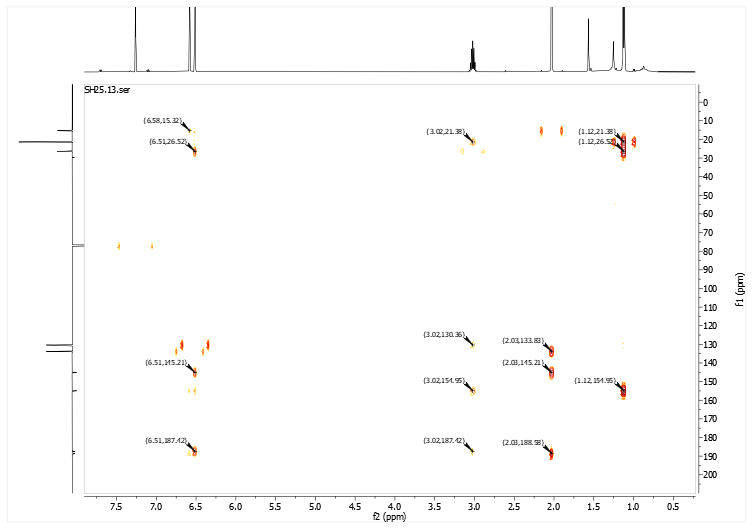


Figure S15. COSY spectrum of compound 2
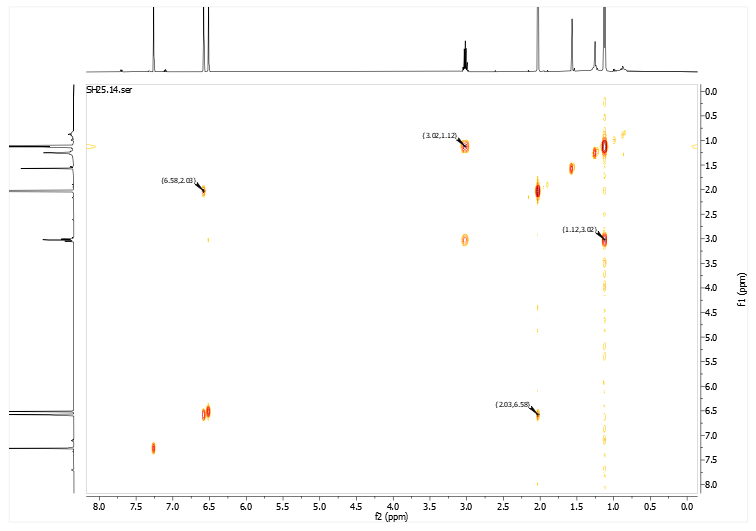

Figure S16. ^1^H-NMR spectrum of compound 3
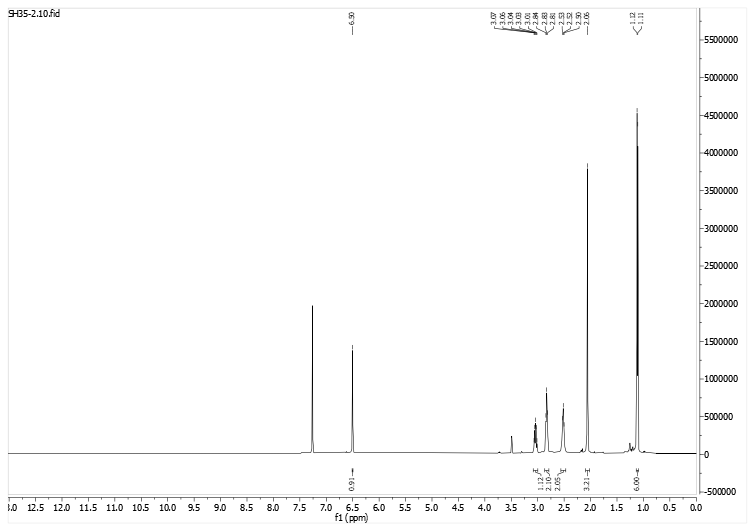


**Figure** S17. ^13^C-NMR spectrum of compound 3
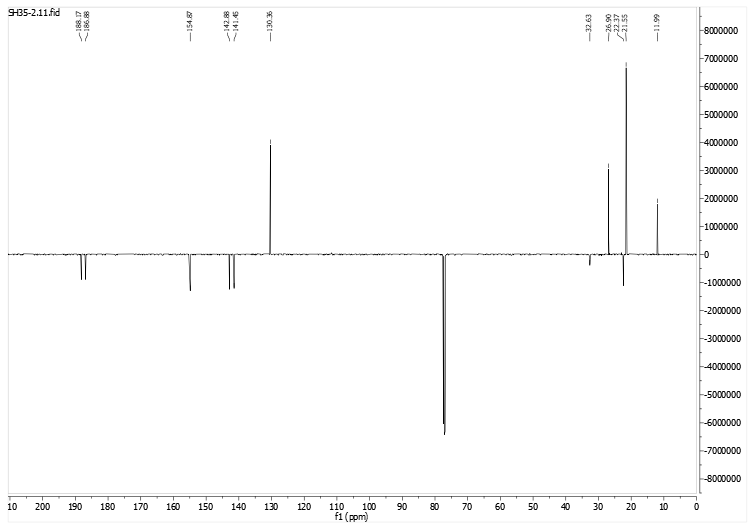


Figure S18. edHSQC spectrum of compound 3
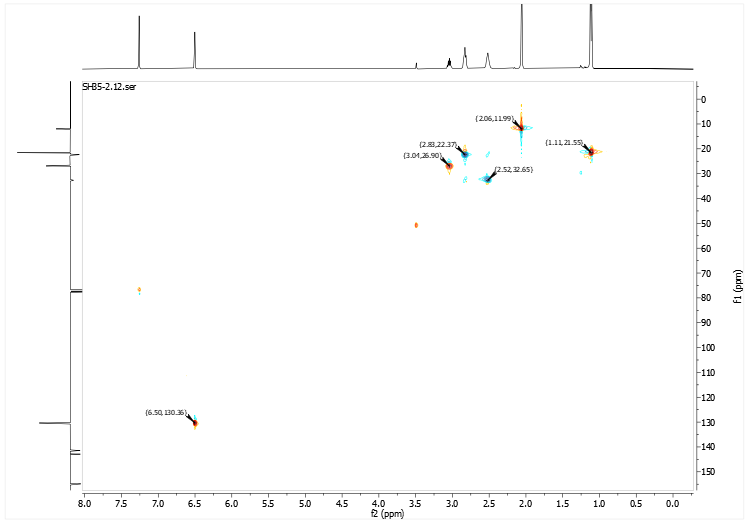


Figure S19. HMBC spectrum of compound 3
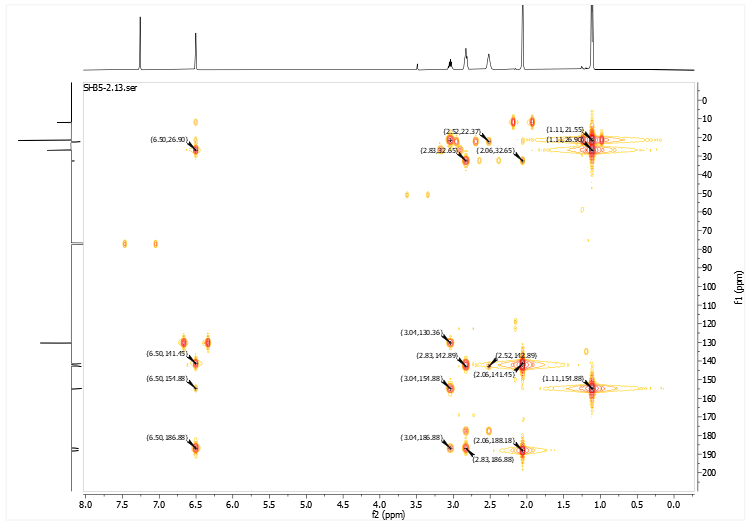


Figure S20. COSY spectrum of compound 3
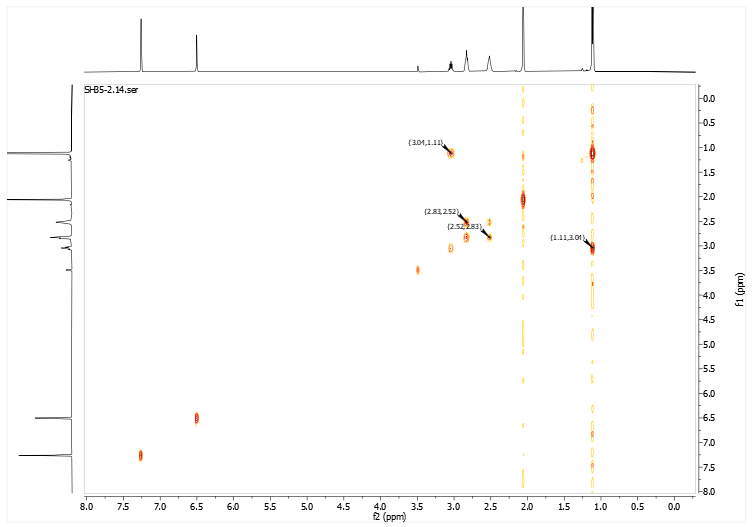

Figure S21. ^1^H-NMR spectrum of compound 4
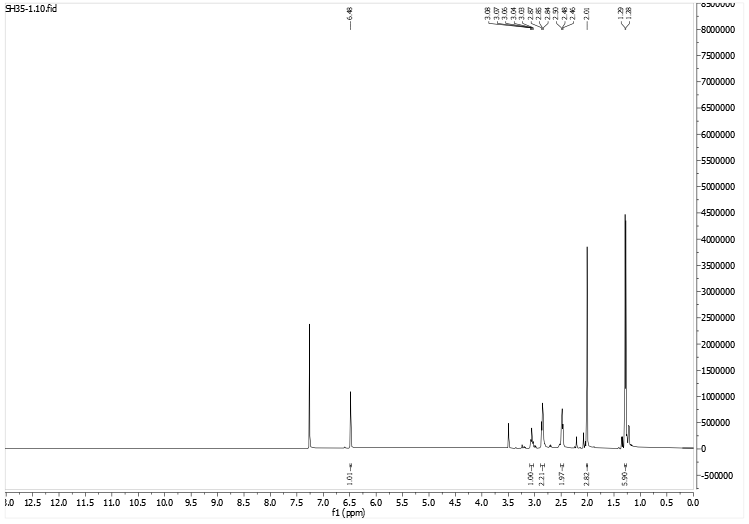


Figure S22. ^13^C-NMR spectrum of compound 4
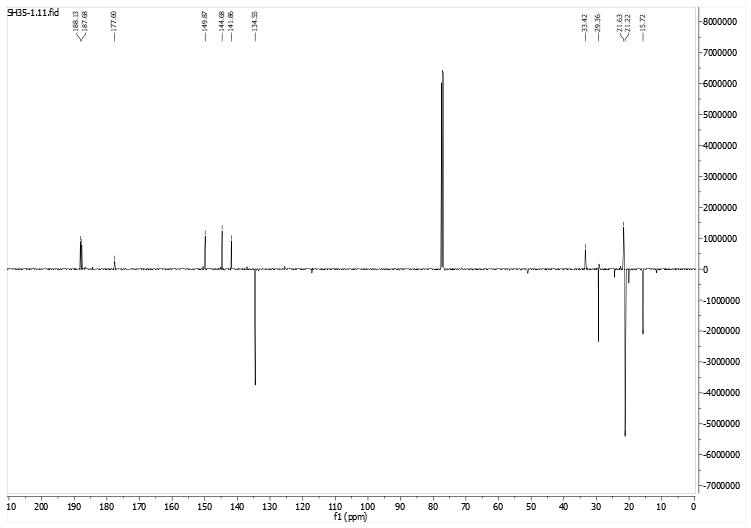


Figure S23. edHSQC spectrum of compound 4
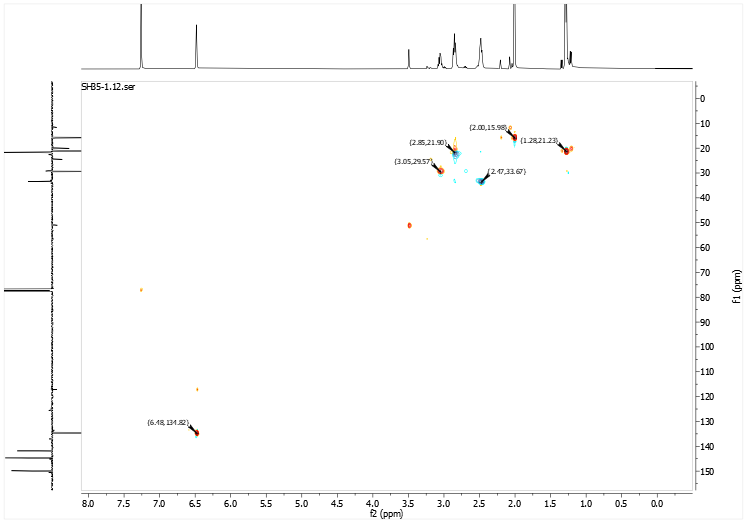


Figure S24. HMBC spectrum of compound 4
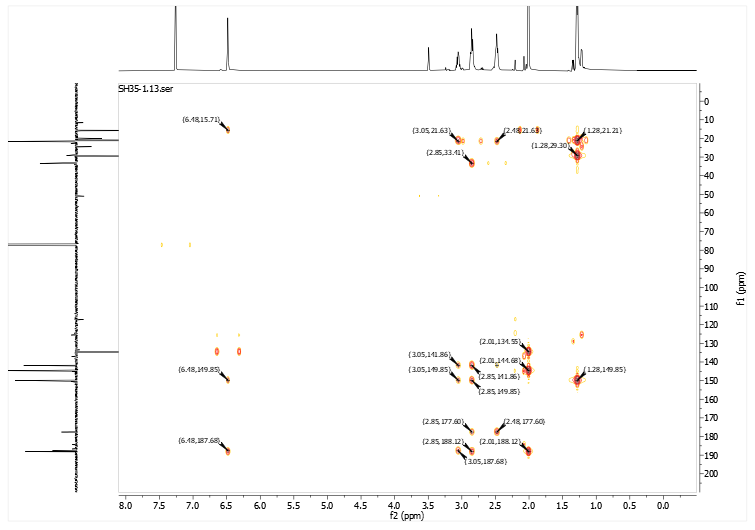


Figure S25. COSY spectrum of compound 4
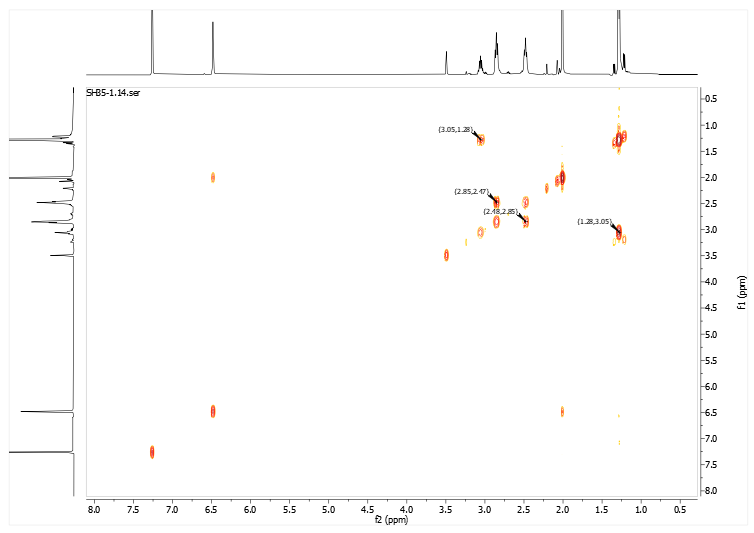

Figure S26. ^1^H-NMR spectrum of compound 10
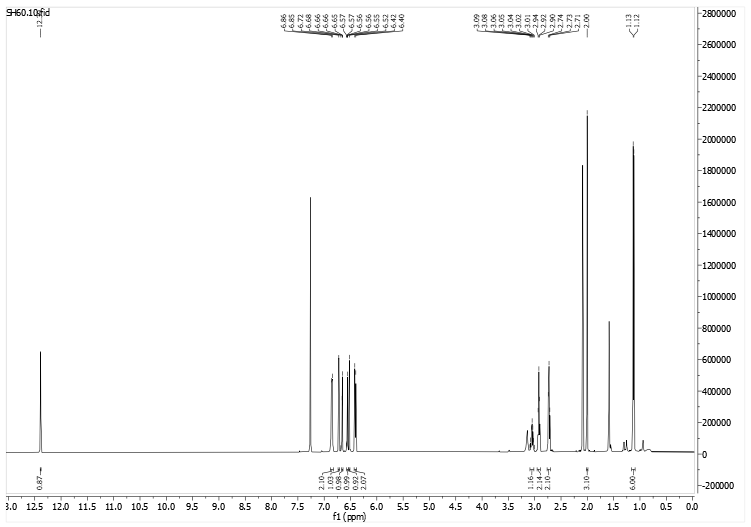


*****

* Extra peak for AcN solvent used during compound preperation is visible in this compound spectra and some of the following compounds.

Figure S27. ^13^C-NMR spectrum of compound 10
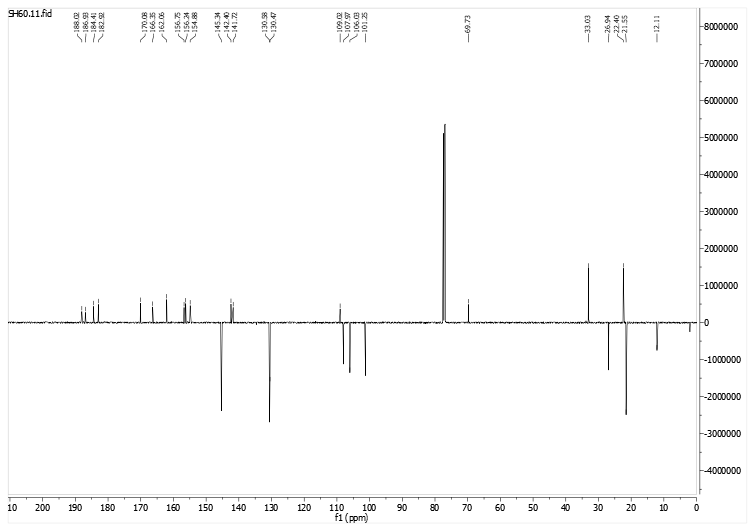


Figure S28. edHSQC spectrum of compound 10
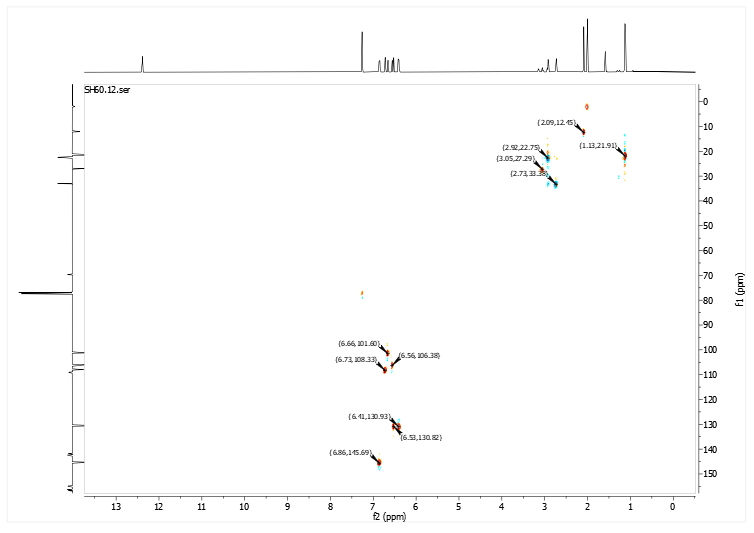


Figure S29. HMBC spectrum of compound 10
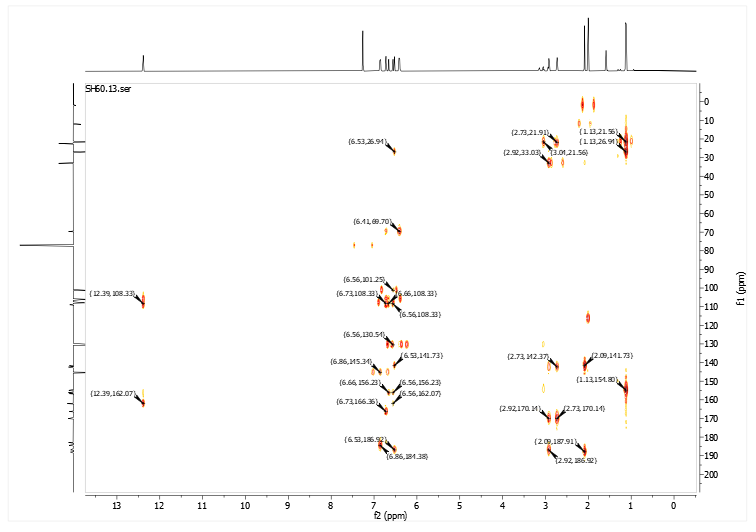


Figure S30. COSY spectrum of compound 10
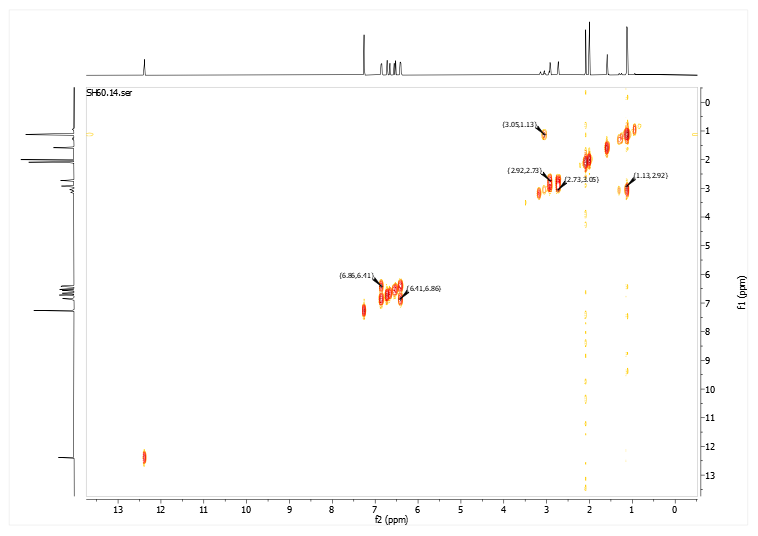


Figure S31. ^1^H-NMR spectrum of compound 11


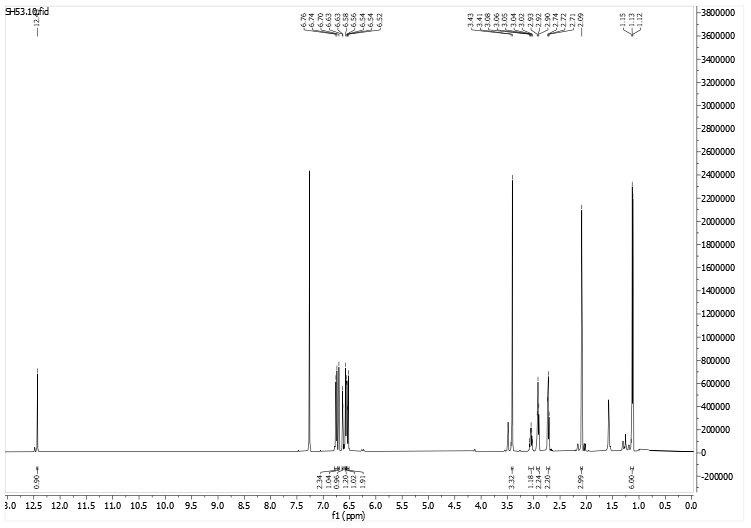


Figure S32. ^13^C-NMR spectrum of compound 11
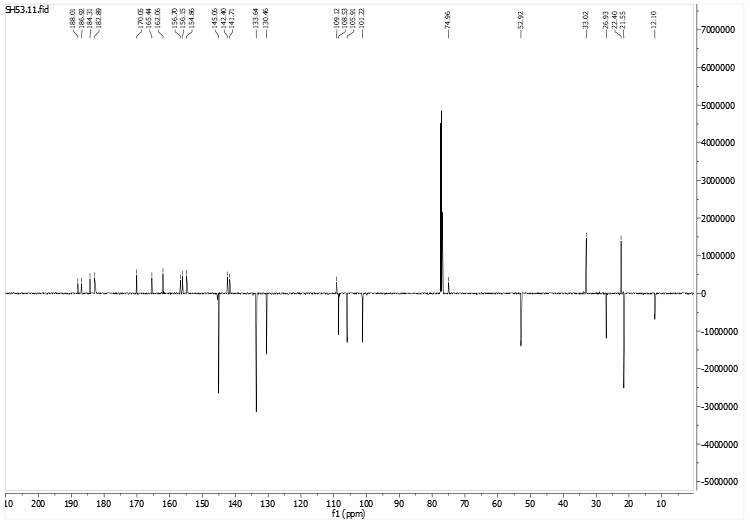


Figure S33. edHSQC spectrum of compound 11
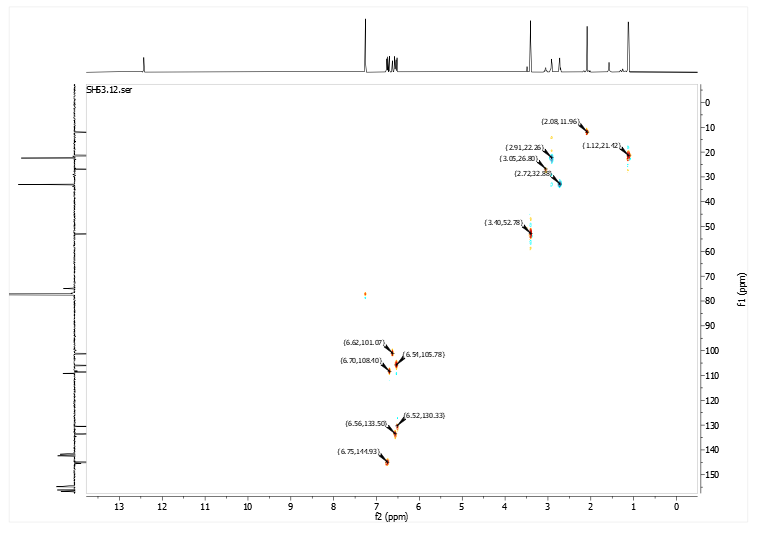


Figure S34. HMBC spectrum of compound 11
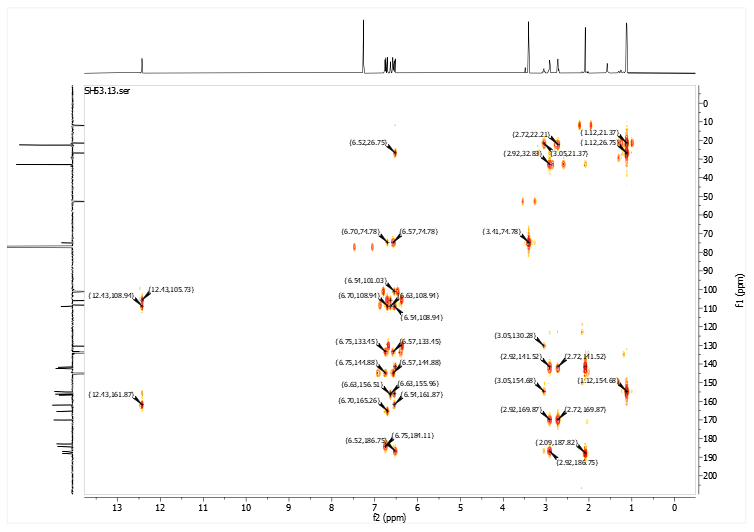


Figure S35. COSY spectrum of compound 11
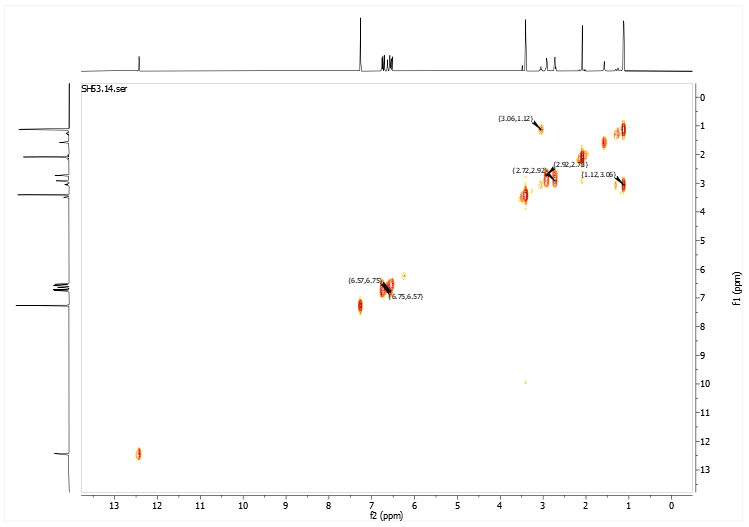

Figure S36. ^1^H-NMR spectrum of compound 12
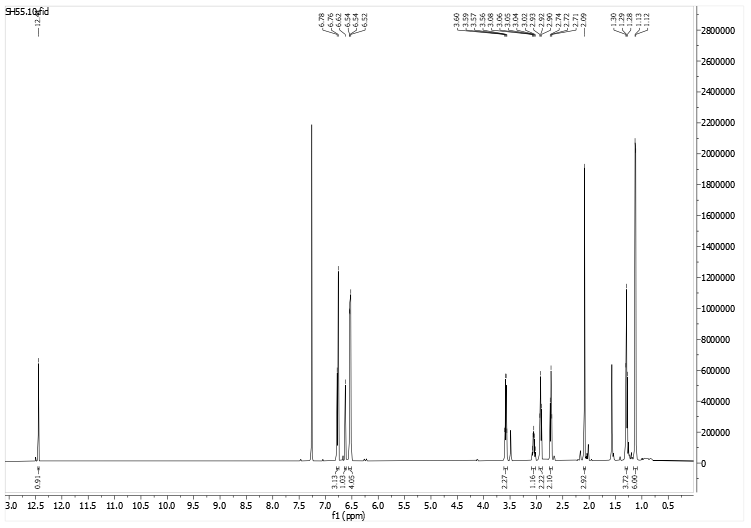


Figure S37. ^13^C-NMR spectrum of compound 12
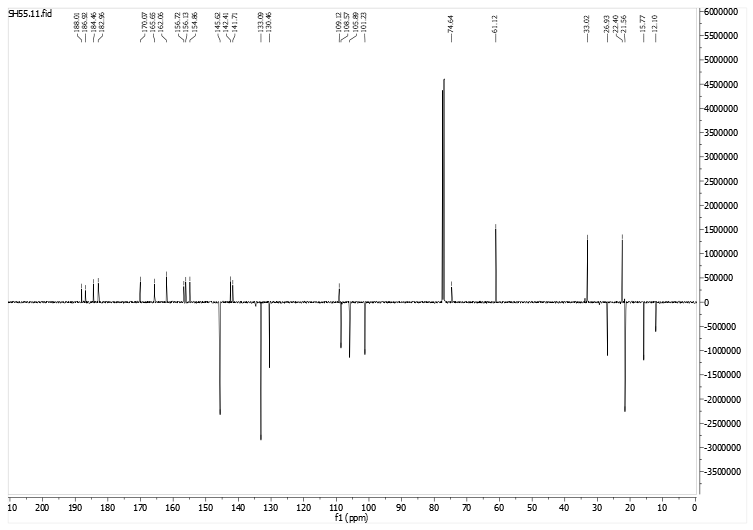


Figure S38. edHSQC spectrum of compound 12
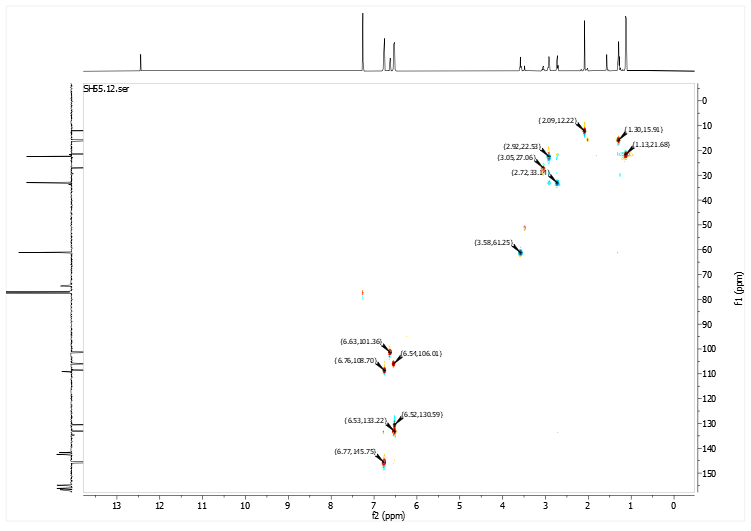


Figure S39. HMBC spectrum of compound 12
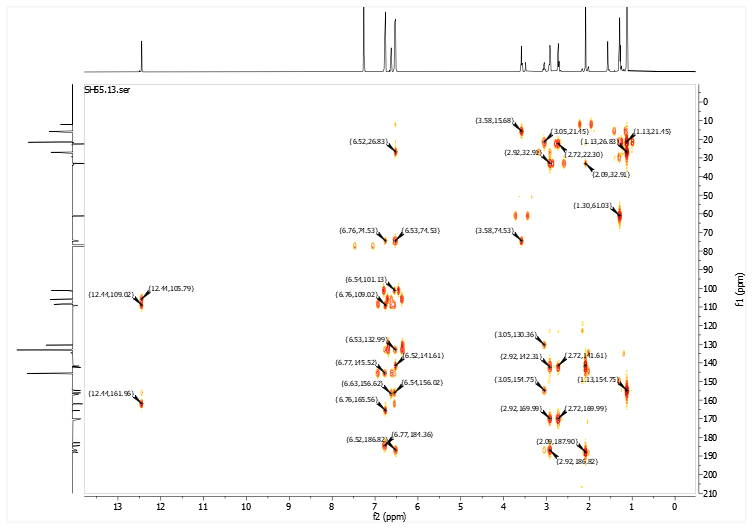


Figure S40. COSY spectrum of compound 12
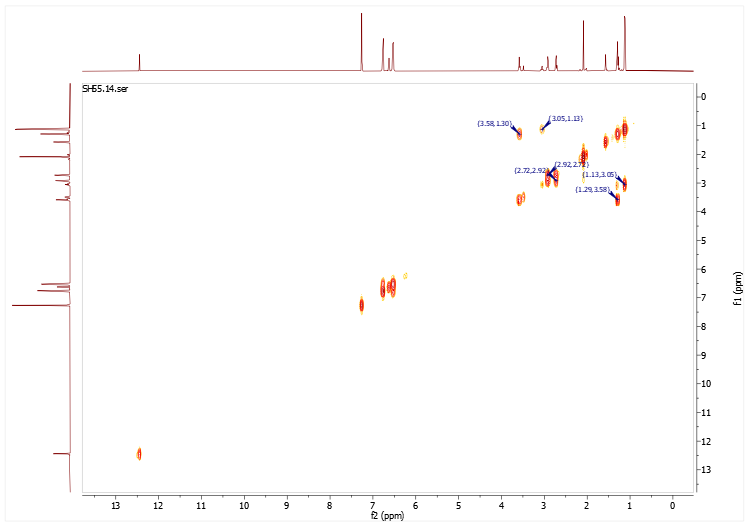


Figure S41. ^1^H-NMR spectrum of compound 13


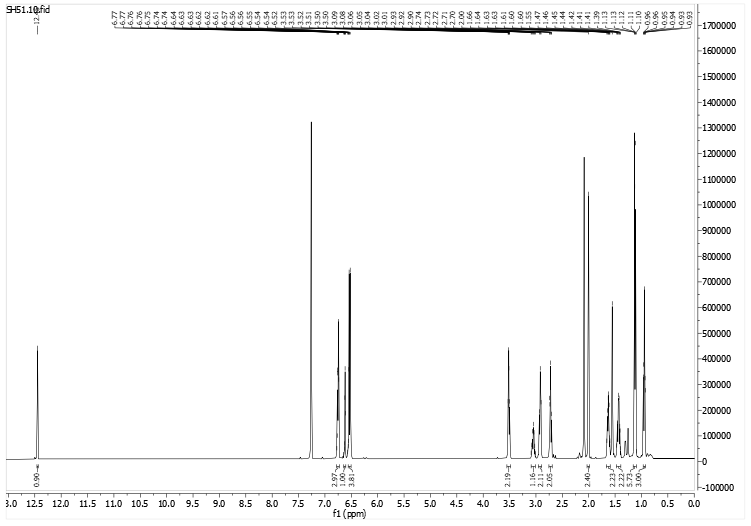


Figure S42. ^13^C-NMR spectrum of compound 13


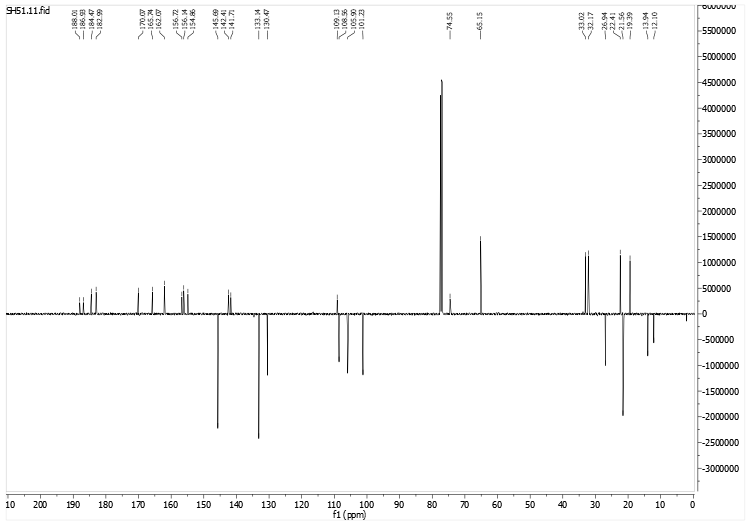


Figure S43. edHSQC spectrum of compound 13


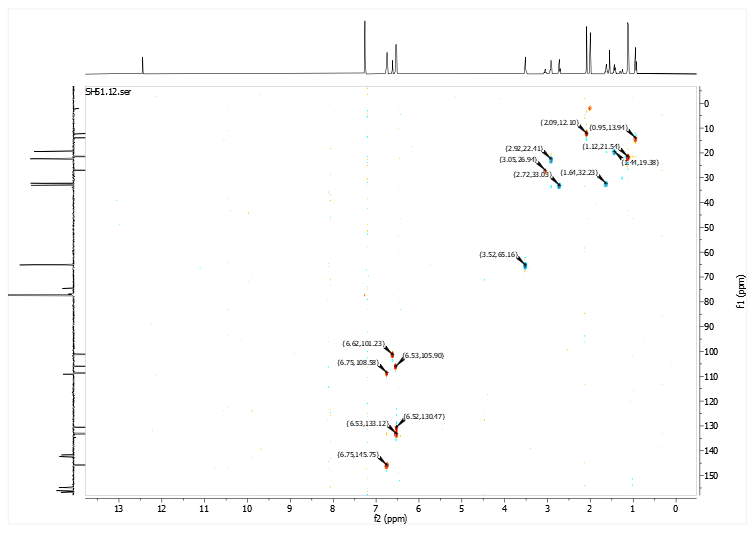


Figure S44. HMBC spectrum of compound 13


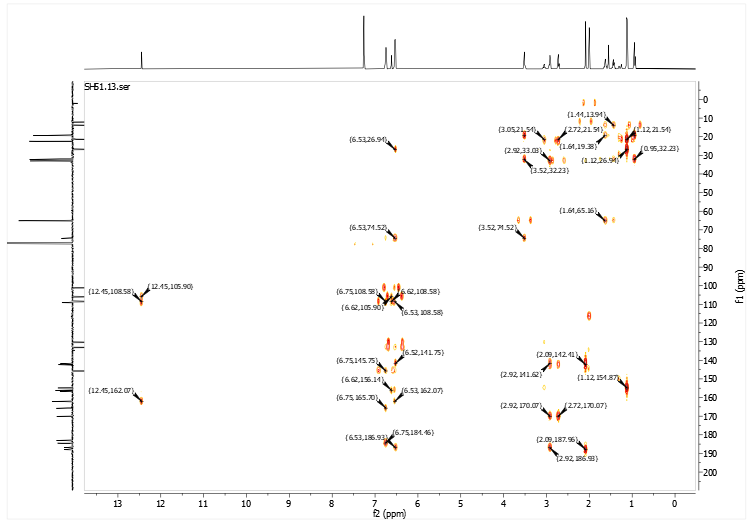


Figure S45. COSY spectrum of compound 13


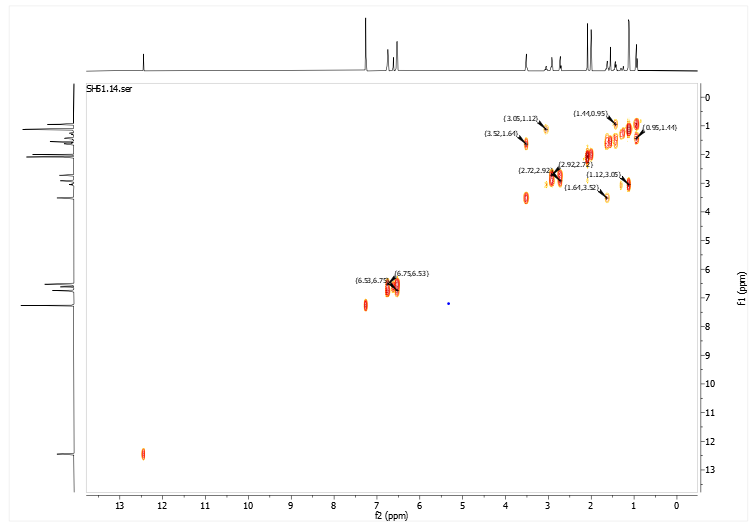


Figure S46. ^1^H-NMR spectrum of compound 14


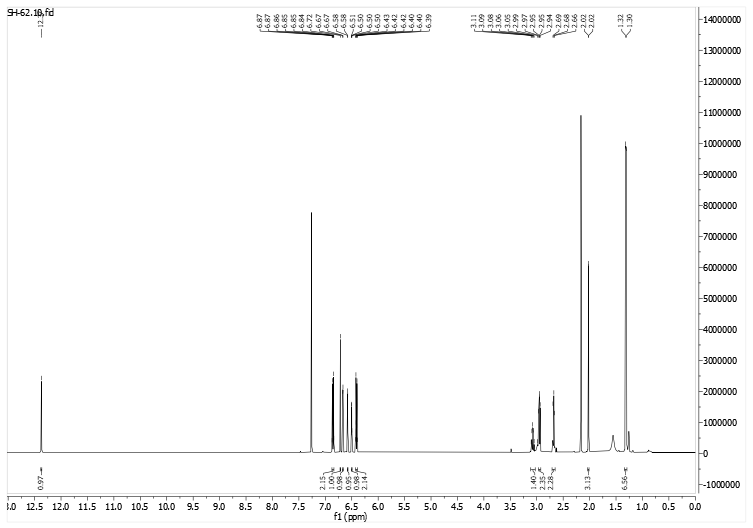


Figure S47. ^13^C-NMR spectrum of compound 14
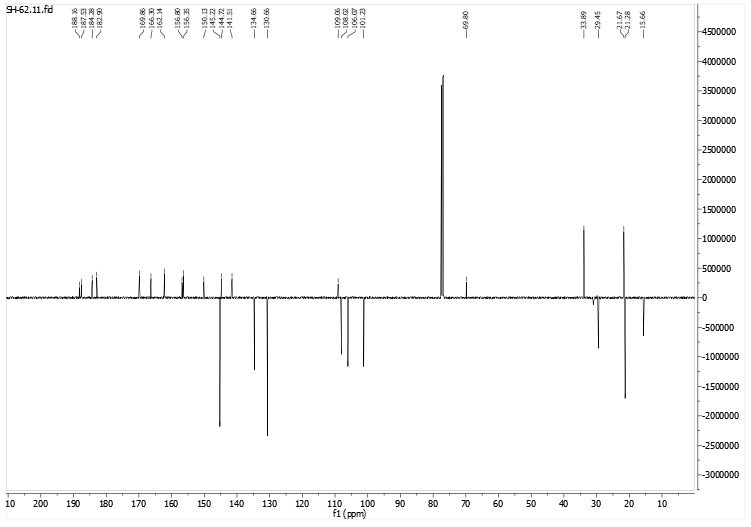


Figure S48. edHSQC spectrum of compound 14
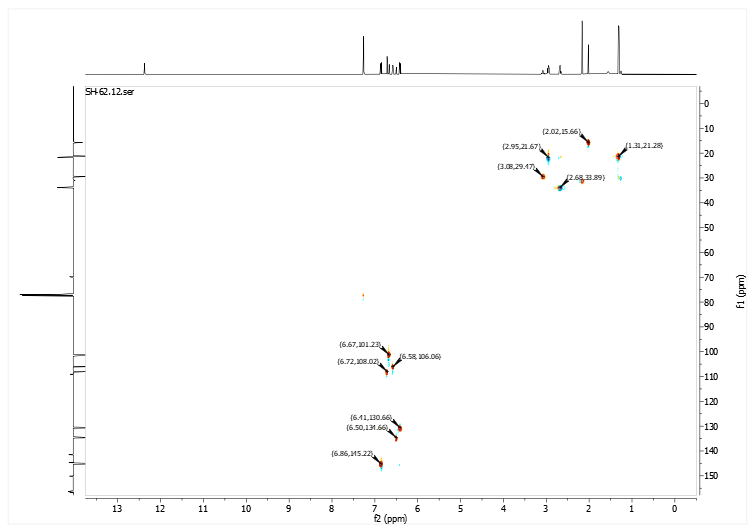


Figure S49. HMBC spectrum of compound 14
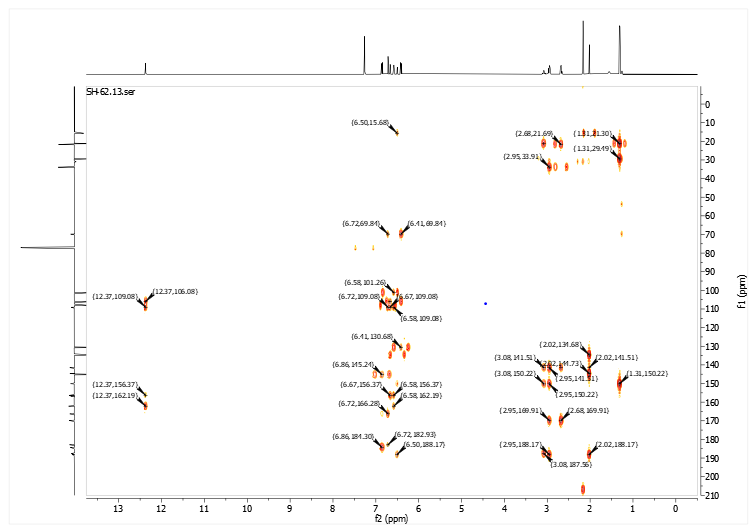


Figure S50. COSY spectrum of compound 14


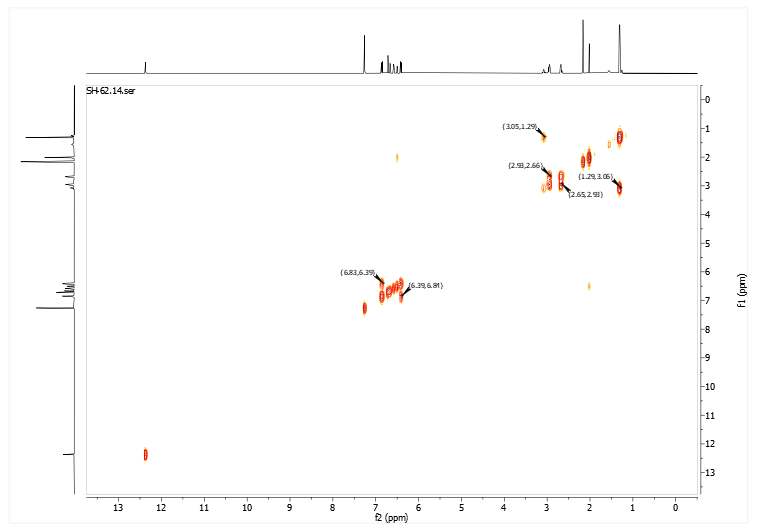


Figure S51. ^1^H-NMR spectrum of compound 15


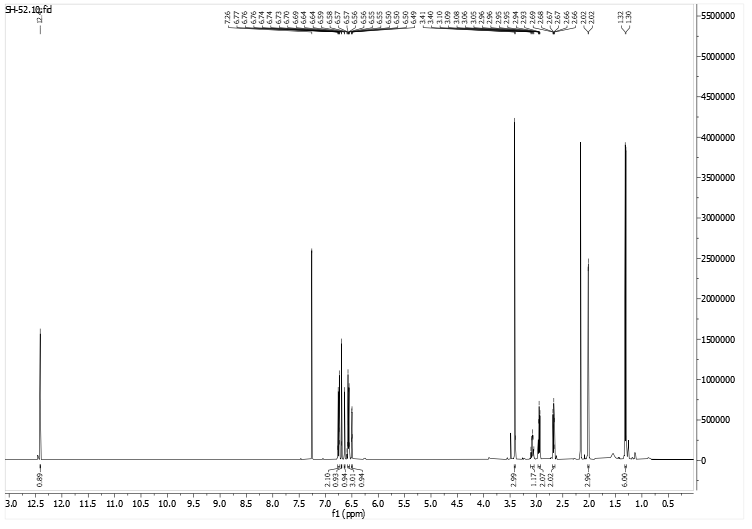


Figure S52. ^13^C-NMR spectrum of compound 15
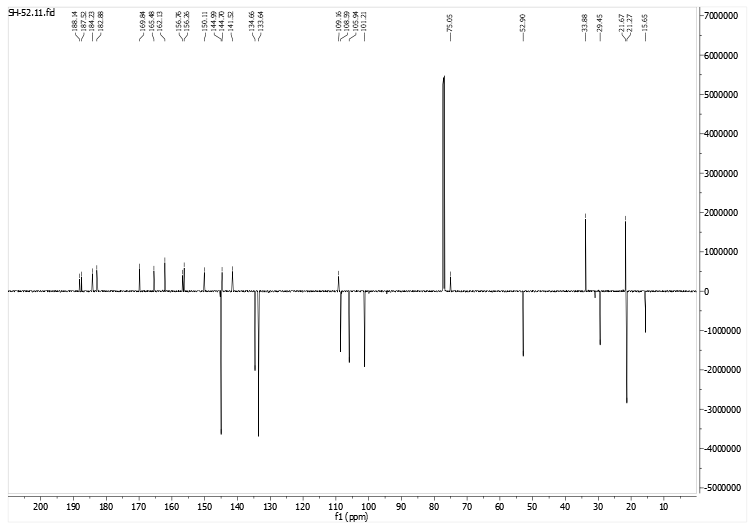


Figure S53. edHSQC spectrum of compound 15
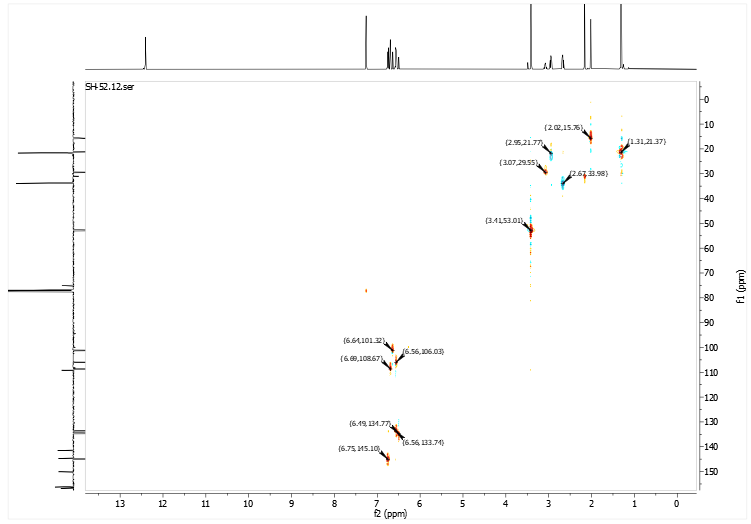


Figure S54. HMBC spectrum of compound 15
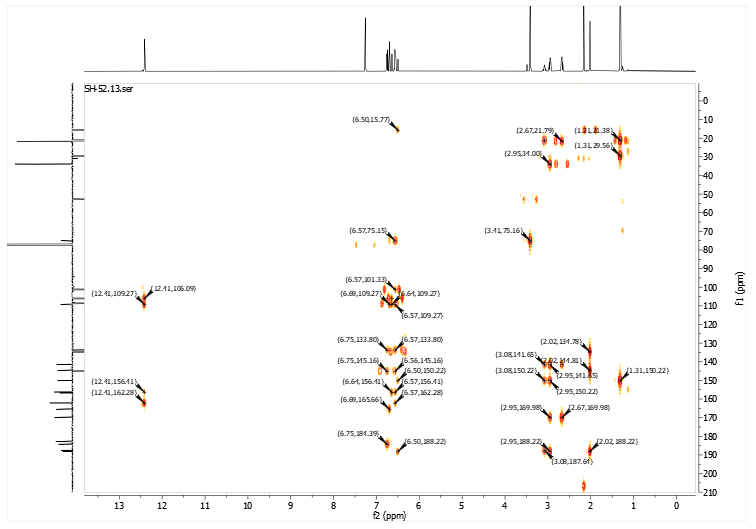


Figure S55. COSY spectrum of compound 15
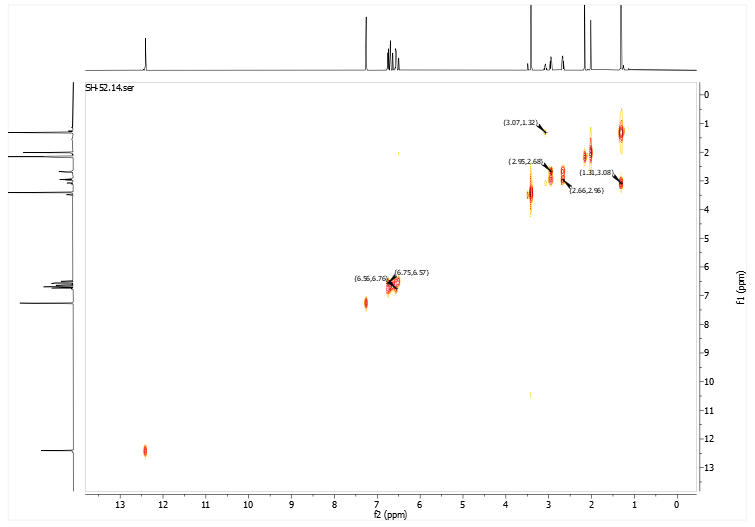


Figure S56. ^1^H-NMR spectrum of compound 16


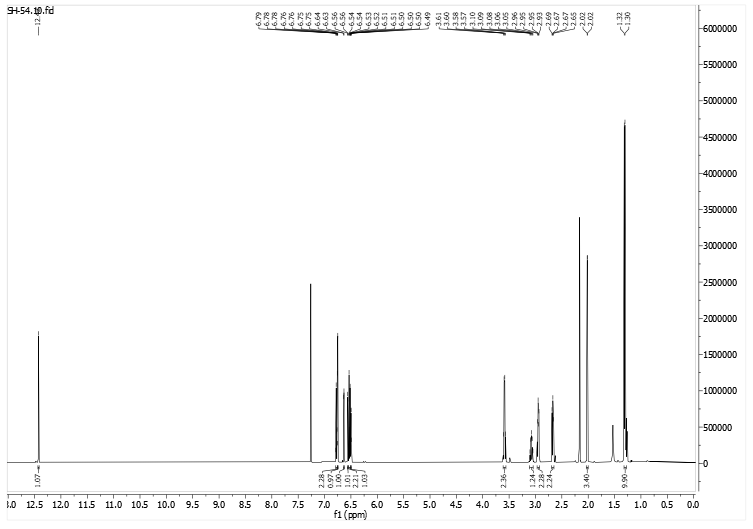


Figure S57. ^13^C-NMR spectrum of compound 16
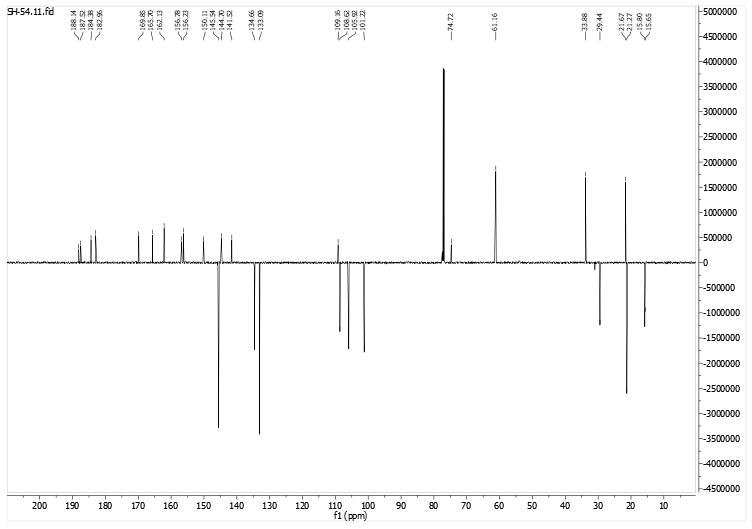


Figure S58. edHSQC spectrum of compound 16
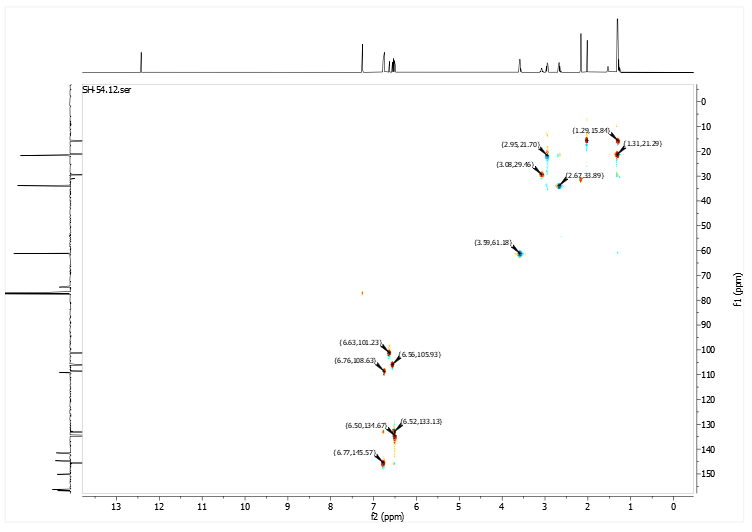


Figure S59. HMBC spectrum of compound 16
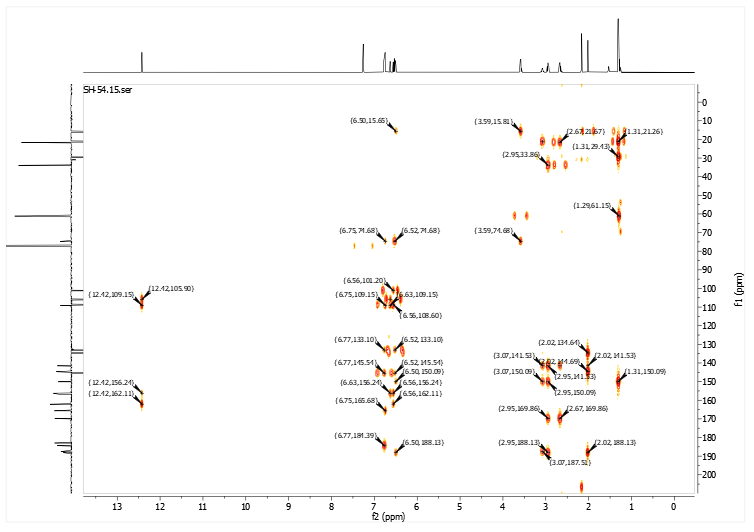


Figure S60. COSY spectrum of compound 16
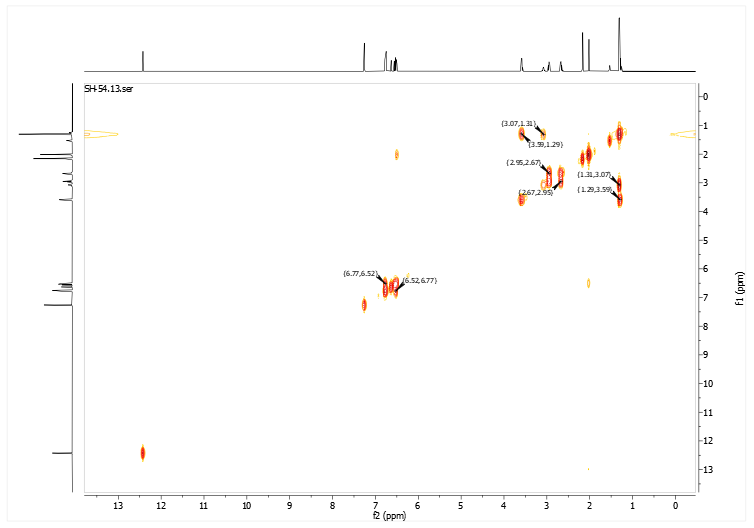


Figure S61. ^1^H-NMR spectrum of compound 17


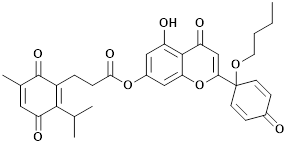

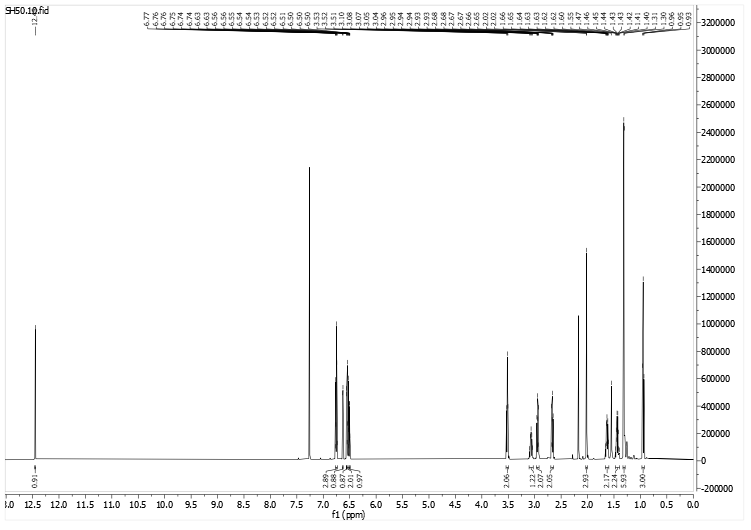


Figure S62. ^13^C-NMR spectrum of compound 17
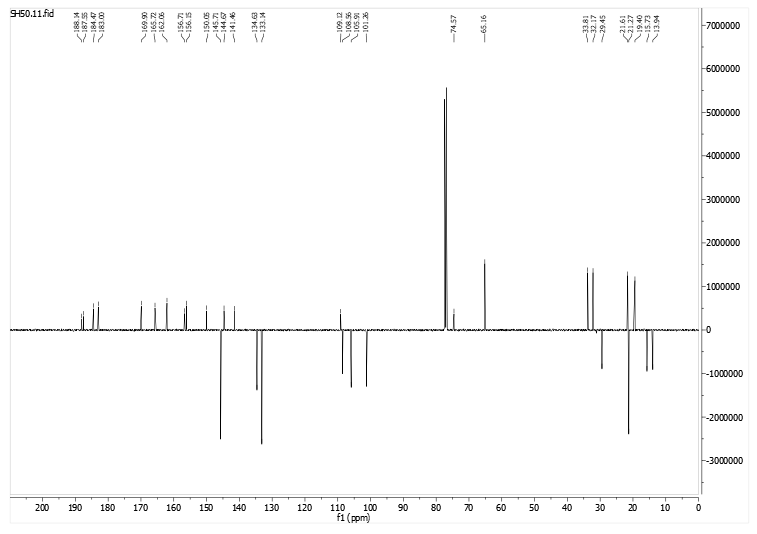


Figure S63. edHSQC spectrum of compound 17
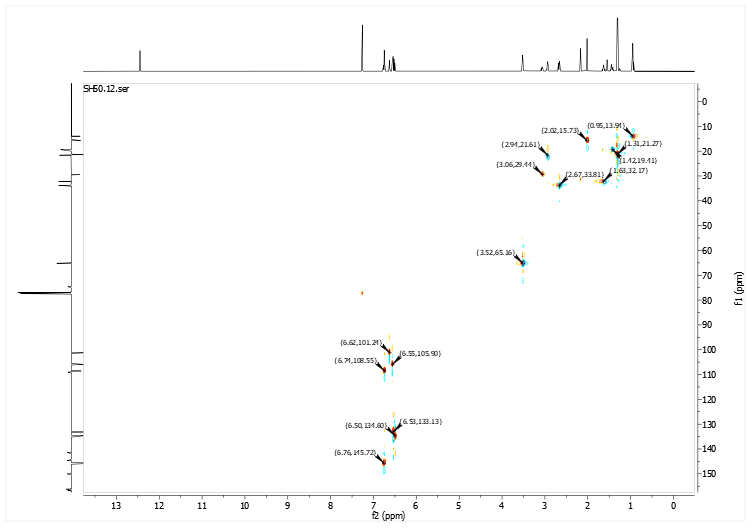


Figure S64. HMBC spectrum of compound 17
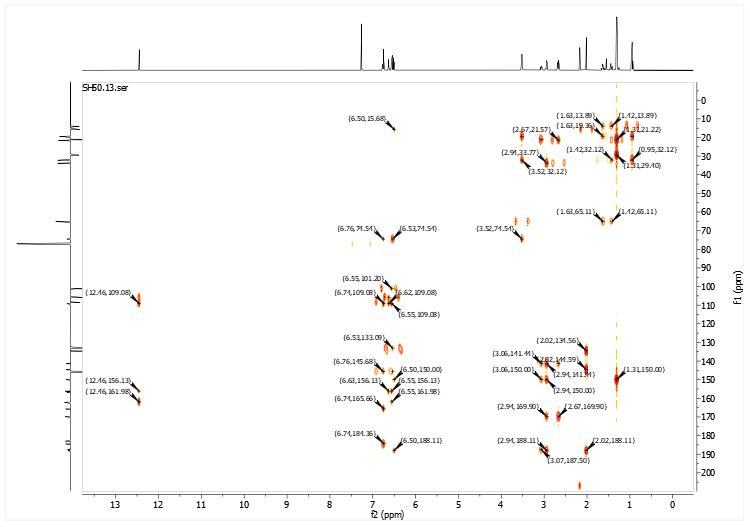


Figure S65. COSY spectrum of compound 17
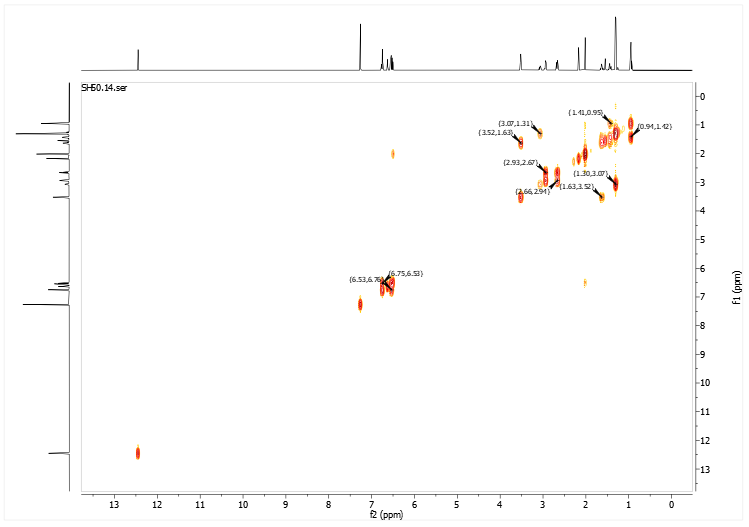

Supplement: S1 File — (DOCX) [file pone.0291567.s001.docx]
